# The Voluntary Hospital: On Its Trial

**Published:** 1914-06-27

**Authors:** G. H. Hume

**Affiliations:** Vice-President Royal Victoria Infirmary


### THE VOLUNTARY HOSPITAL: ON ITS TRIAL.

**By G. H. HUME, M.D., F.R.C.S. Ed., D.C.L., Vice-President Royal Victoria Infirmary.**

I propose in this paper to inquire into the working of the voluntary system in the support of hospitals in the past and at the present time, and to base on this inquiry a forecast as to the future. It may be well at the outset to define the sense in which I use the phrase "on its trial." I mean the phrase to imply that the methods of hospital support which prevail in this country are being put to the proof and tested as regards their stability and efficiency in a way which has not hitherto been realised. This rigorous proof arises partly from certain characteristics which are inherent in the system; in greater measure from developments in our social life which have been long in progress but of late years have been coming rapidly to a head.

In a comparatively recent speech at the Leicester Royal Infirmary, Sir Henry Burdett said, "The voluntary system of hospitals is one of the glories of the world." Few who have gone into the matter, either generally or in connection with the history of some particular hospital, will think this language exaggerated. It is an achievement which has been sustained for now nearly two centuries, and of which our country has good reason to be proud. But in its more intimate working, as regards its drawbacks as well as its glories, it well repays examination; and, indeed, this examination is forced upon us. Circumstances have changed and are changing rapidly, and the changing conditions have brought into question, among many other problems, our method of hospital administration. There is a belief, as much in the mind of the general public as of hospital managers, that our methods call for readjustment. I take it that one of the functions of this Association is to examine such fundamental problems, and by consideration and discussion to form a body of opinion which shall influence coming decisions.

What the voluntary system has accomplished in the founding and support of hospitals is best studied in the history of one of the old institutions; and naturally I turn to that with which I am familiar—the hospital in which you are met. It began more than a century and a-half ago, when so many hospitals were started throughout the country. It is usual to say that they had their origin in a wave of philanthropy; it is certainly true that a wonderful well-spring of charity was then flowing into many channels, and chief among these into a new interest in the sick and suffering poor. There was a healthy rivalry among the great towns to be forward in the hospital movement, and the writer of a famous letter which led to the founding of this infirmary taunted his fellow-townsmen with being slow to follow the laudable example of others. A movement begun in this way was sure of success, and there seems to have been little difficulty in any case in getting money for hospital building and subsequent support so long as the wave of enthusiasm lasted. But waves of enthusiasm have their ebb as well as their flow. In ten years the Newcastle Infirmary had accumulated an invested capital of £7,000 from its annual surpluses. In thirty more years the tide had ebbed and the infirmary found itself in very low water indeed. Public interest was well-nigh dead, the infirmary was indebted to its treasurer, and as both effect and cause the hospital had become inadequate and inefficient. Then, when things were at their worst, they began to mend. Interest was revived chiefly, or at any rate largely, because the man (Dr. John Clark, physician to the infirmary) arose for the occasion, and with much labour won the public to a knowledge of the facts and of their own responsibility. The infirmary was reconstructed and enlarged, and with increasing resources found itself for a time equal to its ever-growing task. The same cycle of events has twice since that time recurred in the history of our infirmary. Forty years after this first reconstruction in 1801, it began to be realised that it had again fallen into an inefficient state, and that it had been allowed to lag far behind what the amazing expansion of the town and district in these years required. Small blame, indeed, to the Governors of the infirmary that their institution had failed to keep pace with this phenomenal growth. But they had apparently not tried. It took ten more years to arouse the public mind, and it was not until 1855 that a substantial addition to the hospital was inaugurated. There followed, as had done before, revived interest, increased resources, and efficient work. The quarter of a century which ensued was one of the most prosperous periods financially that the institution has had. As an indication the invested capital increased from £14,000 in 1853 to £58,000 in 1876. Then came again dark days, years of financial strain, of unpopularity and much hostile criticism, and of a growing sense of inefficiency. The tide was slow to move. The undertaking which the Governors had this time to face was on a grander scale; nothing short of a new hospital on a new site, it was recognised, would meet the needs of the district. After prolonged discussion a make-shift was adopted. In 1885 a temporary block was opened which was intended to last for only five years, so as to give time for the larger task to be undertaken and possibly carried through. Instead, it lasted and continued in use with the old infirmary for twenty-one years. The tide had taken ten years before it began to move, and ten more years were to pass before the task of building a new hospital was complete. It is possible—indeed, it is probable—that the delay would have lasted longer but for the happy conjunction of the occasion and the man. The occasion which touched the heart of the public was the commemoration of the Diamond Jubilee of Queen Victoria; and it was rendered fruitful by the proposal of the then Mayor (now Sir Riley Lord) that Newcastle's tribute to the Queen should be a new infirmary. The result was the hospital in which you are assembled.

I have ventured to obtrude upon you these facts in the history of a local hospital for a purpose. In the first place, I offer them as in no way exceptional. The same experiences have been gone through, and the same task has been as nobly accomplished, wherever throughout the country voluntary hospitals have been established and maintained. And they justify, surely, the dictum of Sir Henry Burdett which I have already quoted. But for our consideration at present and for the purpose of discussion the details which I have cited illustrate some of the serious drawbacks of an exclusive reliance on voluntary support in the provision and maintenance of hospitals. These drawbacks are mainly two—namely, the fitful and uncertain character of voluntary giving; and, secondly, its often tardy response to special needs, as when reconstruction or enlargement are urgently demanded in the interest of the sick and of the public. My sketch will help you to realise how aptly the phrase "a wave of philanthropy or charity" fits the facts. Voluntary giving is too largely dependent on emotion, on varying interest, on a thousand and one shifting circumstances and influences, to have been or to be an entirely reliable support for hospital work.

Another truth which stands out from my brief survey of local experience, and which I incidentally emphasise, is that inefficiency in the hospital is often itself the source of public apathy. It has always been true that timidity is the worst fault in hospital administration. A confident doing of the best work possible is the only way to command a willing support.

I now pass on to consider the changed and changing circumstances to which I have referred, and which to many suggest doubt as to the continuance of present methods. The first is a change of attitude on the part of both givers and recipients. The giver is probably less conscious than his early predecessor of the charitable emotion as the incentive to his giving. The recipient is more disposed to regard what he considers his rights, but is also, let it be thankfully acknowledged, more alert to practise the useful virtue of self-help. It would perhaps be just to say that an enlightened self-interest has become operative on both sides, and herein is undoubtedly a ground of encouragement for the future. The industrial classes are themselves becoming actively interested in the welfare of the institutions on which they depend for help in disablement or serious sickness. And we think, in Newcastle, that as an organised movement this participation of the working classes began here. It has at least reached its furthest development here, so that the sum of £20,000 or thereabout gathered annually from this source is now the largest coherent item in the hospital income. Needless to say, it has brought in its train important changes and adjustments in the constitution of the infirmary; but the new influence has been mainly to the good. Besides the much-needed revenue, it has introduced an influence active and progressive, and one that is likely to combat the tendency to periodic stagnation which is apt to invade hospital affairs. But to this gain there is a counterbalancing loss. It becomes a misfortune if the activity and self-denial of one class should blunt or render indifferent the interest of other sections of the community. There is no lack of considerations which ought to correct this tendency. In the first place, one class alone cannot, and ought not to, maintain an institution in which all have a vital concern. Less directly, but no less really, those who are themselves the least likely to be indebted to its care are nevertheless affected by its welfare. We are too closely interlinked with our fellows not from time to time to have some one whose life and health are of moment to us, and whose care in sickness and whose restoration are due to hospital treatment. And the claim on employers of labour is stronger still. It will not be disputed that the health of the workmen is an employer's asset, and in the guarding of it it would be well if both could cordially combine. Nevertheless the growing independence of the classes from whom hospital patients chiefly come, and its natural result in the decline of interest among those who have hitherto been hospital supporters, is largely the source of present difficulties. But most of all, in the general opinion, is the position undergoing change through the tendencies of recent legislation, more especially through the operation of the Insurance Act. The working of the Act is responsible for much of the increasing pressure. This has all along been anticipated, though it is not quite easy to put the finger on the precise modes in which it is so acting. Of one factor we may be quite sure. Many, especially of the very poor, who were never able to see a doctor before, now that they avail themselves of their new right to medical attendance are found to be the subjects of ailments which call for hospital treatment. The attendances at the panel doctor's surgery are taking the place to a great extent of the hospital out-patient department as it was; and they are acting more efficiently in discovering cases fit for or requiring in-patient treatment. The Act itself makes no attempt to supply this increased demand which it has helped to create. Section 21, which authorises approved societies to contribute to the support of hospitals, remains a dead letter and is hardly even a pious wish. In their present and threatened financial position approved societies are hardly likely to avail themselves of the permission to contribute. Without seeking the concurrence of the voluntary hospitals, the framers of the Act have assumed their readiness as well as their ability to continue their old work on an enlarged scale. Yet without them the National Health scheme is wholly incomplete. It is not an unfair criticism of the Act to say that it provides mainly for the treatment of trivial ailments while leaving the serious cases to be dealt with as they can by the voluntary hospitals. The panel doctor is instructed that he is responsible only for treatment "of a kind which can, consistently with the best interests of the patient, be properly undertaken by a practitioner of ordinary competence and skill." That is, he is not called upon or expected to do surgical operations nor expert work of any kind. In cases requiring such his responsibility ends when he has advised the patient where he or she is to seek the help needed—that is, as a rule, if the case be not a tuberculous one, to seek admission to a voluntary hospital. And the hospital may be one which is hampered by a growing waiting-list, and is unable without long delay to admit any but urgent cases and cases of emergency. That is the burden which the Insurance Act tacitly throws on the voluntary hospitals.

But more subtle in its working, and even more important than this increase of hospital pressure, is the effect which the Insurance scheme must have in precipitating the change of mutual attitude of givers and recipients. The scheme holds out in theory at all events the promise of an efficient medical and surgical assistance in return for payments made. It practically acknowledges that right to claim what was once a matter of charity. And the change of attitude is completed when the hospital looks to the recipients of hospital benefits for substantial hospital support.

No less undoubted and showing itself in a very practical way is the change of attitude on the part of the givers. The converse of the same causes has been at work upon them. It is showing itself in a growing reluctance to give. And, further, if we would duly appreciate what this means we will candidly admit that it has never—to omit for the moment those brief times of enthusiasm to which I have referred—it has never been an easy task to keep a hospital going. Looking back over the past we are struck by the magnitude and the splendour of the work that has been done. We lose in our retrospect, or we take little account of, the labour that it cost and of the obstacles that never granted more than a partial or temporary success. Some years ago, with an object in view, I set myself to read through every page of the minute-books of this infirmary. This laborious perusal left on my mind two deep impressions. The first was an admiring sense of the fidelity and constancy of those who through the long succession of years administered the affairs of the charity. But with this there was a sense of continuous and often unfruitful effort which must have chilled, and did often chill, the energies of the workers. There was a third impression which has become a conviction in my mind and which I venture to express to-day. I am convinced that the voluntary system will fail to meet with anything like adequacy the approaching demand for increased hospital accommodation.

I have said that there has of late shown itself a growing reluctance to give to hospitals; and it is my duty to bring forward evidence in support of this statement. We find it in the annual reports of nearly all the hospitals. There are still a fortunate few who can congratulate themselves on their year's working and can also report a favourable balance in the year's finances. But it is a significant fact that the complaints of deficiency come mainly from the large centres of population. There naturally one expects the greatest pressure, but that is not the whole truth. The decline of interest in hospital work and the conflicting interests of the different sections of the public are more responsible than the increasing needs of the community. Increase of population and consequent increase of demand for hospital accommodation are for the most part attended by a corresponding increase of wealth; and if this brought with it also an increase in the will to give, the solution of the problem would not be far to seek. But, on the contrary, if we take the ordinary voluntary income as a gauge of the level of public interest in hospital work, we find in the experience of most hospitals that this has for many years been either at a standstill or has increased in no proportional relation to the amount of work done. Let us take the experience of two hospitals in two of the first cities in the kingdom in illustration. The annual report of the Royal Southern Hospital of Liverpool, after showing the increasing disparity between the total ordinary income and expenditure for the last seven years, goes on to say: "This ever-increasing working deficit gives the committee much alarm, and if the work and efficiency of the hospital is to be maintained, it will be necessary for the public, especially the working classes who receive its benefits, to realise that this can only be done by increased support." More striking still is the experience of the Royal Infirmary of Manchester. I quote from the *Times* notice, with comments, of the annual meeting of the Trustees. After giving the figures for the year 1913, which show a deficit on the working of the Royal Infirmary and Convalescent Home of £5,471, the report goes on: "What makes the matter even worse is that the ordinary voluntary income last year only amounts to £15,320, the remainder of the income being derived from investments, patients' contributions, the absorption of all available legacies, and by encroachment on the capital." And the comment is added "that when the immense volume of business and wealth of Manchester is considered £15,320 does not seem to be an adequate sum to be allotted to Manchester's premier charity." I have purposely cited these two hospitals as witnesses because they belong to great and immensely wealthy communities. But their experience is in degree the experience of all, and the conclusion cannot be escaped that the voluntary subscriber has become lukewarm in his support of hospital work, that he has been influenced by the change of position and attitude on the part of the recipients of hospital benefits, and that he has been made distrustful of the future by the trend of recent legislation.

Again, it is easy to find authorities in support of the opinion as to the general decline of voluntary support, more marked if this be taken as meaning the proportional decline. The Secretary of the London Hospital, Mr. E. W. Morris, whose most interesting history of his hospital should be read by everyone, says, "Most hospital secretaries will own that to keep up their subscription lists becomes a harder and ever harder struggle." At the meeting of this Association at Oxford last year there was no lack of similar opinion expressed in the discussion on the effect on hospitals of the Insurance Act. The Chairman of the Council, Dr. Macintosh, said, "The country must either go to the State for support or the voluntary system must receive more enthusiasm than in the past." Mr. Alvey, of Charing Cross Hospital, "thought that the voluntary system would have to be considerably modified in the future, not only on account of the Insurance Act but of the whole trend of things." In the proposals of other important speakers for the reinforcement of the voluntary system the admission of its present inadequacy was implicit. Some of the proposals I shall presently consider. It is sufficient to say now that everywhere there is a belief that some change is necessary and that it is imminent.

Before I go on to consider this change and the practical measures it will entail I wish to pause in order to direct attention to a subject which perhaps does not fall directly into my line of argument. It is, however, of great importance, and is involved in the financial competence of the hospitals. I would describe it as the scientific standard which the hospitals are enabled to aim at or attain in the character of their work. Necessarily it is not merely a question of endeavour, it is also a question of funds. The advances in all departments of medical and surgical science have admittedly put an additional strain on the modern hospital. This is a change which has been long in progress, but every day is gaining in momentum. The cost of hospital treatment has been thereby greatly increased, and again the demands made upon the voluntary hospital have been courageously, and for the most part efficiently, met. But when we pass from the increased cost of methods of treatment, especially that involved in the antiseptic treatment of surgical cases, and take into consideration the more recently introduced scientific methods in the investigation of disease, we find that the hospitals are perforce tardily responding. The investigation of individual cases is now largely carried out in the laboratory; one need only instance the comparatively new science of bacteriology and its applications. Hence the need of clinical laboratories; and these require not only ample equipment for the work, but also a trained staff. The applications of electricity also both in investigation and treatment are steadily becoming more extended, and also more costly. And, finally, in the investigation of disease in all its forms and their results a well-equipped pathological laboratory and paid staff are essential in every hospital of importance. Nor can the interests of medical education, and in this regard of the public, be overlooked. Unsurpassed as our hospitals are as clinical schools they are lagging far behind those of other countries in the means of scientific training. The chief reason for this is the want of funds; and it is a question how far this want is likely to be remedied so long as the resources of the hospital are derived from an exclusive reliance on voluntary support.

From this digression I return to the consideration of the main question. It may be safely predicted that in seeking a way out of present difficulties we shall follow our traditional course. We shall endeavour to avoid radical change. We shall seek to reinforce, not to abandon the voluntary principle. In all likelihood we shall be found repeating more or less closely the history of education in this country, with happily the difference that the hospital question does not contain those elements of religious discord which have protracted and so often embittered the education settlement. But it is of more than passing interest to note how closely the progress of education and of the hospital movement down to a certain point followed similar lines. Begun for the most part by individual effort and maintained largely from religious and philanthropic motive, both were left to their own development. Then it was realised that education was too important and too grave an interest to be left to unorganised effort, and the Education Acts followed as the expression of a conviction that organised methods and reliable support were indispensable. One of the leading features of our time is an awakened care for health, not so much as an individual concern, but as a social duty. Repeating what occurred with regard to education, and has also already occurred in several departments of public health, there is a growing conviction that our hospital system is in need of the same conditions of successful working—namely, organised methods and reliable support.

Without further pursuing this parallel let me now pass in review the various proposals which have been made for reinforcing the voluntary system. These proposals fall into two groups: those which are designed to increase revenue, and those which are designed to relieve the hospitals of some part of their work, and consequently of some part of their financial burden.

In his presidential address at Oxford last year, Sir William Osler put tersely the policy which he urged. Speaking of the voluntary system—and I take for granted he had in mind only the exclusive reliance upon it—he said, "Give it up! it is antiquated and out of date, and it is not going to continue." His advice practically amounted to this: Get money where you can!

Especially he urged an acceptance of the principle of taking pay from patients. The policy of taking money where it can be got, contradicting though it does the cherished ideas of a free hospital and charitable giving, is that which with more or less frankness is being generally put in practice. The exaction of payments from patients is being adopted in many hospitals. It cannot be said to have become popular. It is unpopular with committees of management, because it runs violently counter to treasured traditions; it is naturally not popular with patients. But there is in many instances a practical difficulty, and it is a difficulty which will become greater as the working classes more generally become contributors. These contributors when they become in-patients resent the expectation that they should pay again; and the same thing applies to insured persons, whose belief in their right to have all necessary treatment free is at least intelligible. At the same time there are patients admitted who are able, and if the custom were once generally established would be willing, to pay something. To my mind it is as much the duty as it is to the interest of every hospital to obtain such payments; and the example of the Manchester Royal Infirmary is encouraging as to the substantial success of the practice. Last year in that hospital a sum of more than £4,000 was derived from this source. In the case of insured persons the hospitals have an undoubted claim to be refunded, and provision for this either as a lump sum or a *pro rata* payment should find a place in an Amending Act.

The better organisation of methods of obtaining subscriptions from all classes—the industrial, the employing, and the general public—is the most obvious and the most difficult of all devices. The working classes, who in this matter are largely self-organised, are in many parts of the country setting the example, and it does not seem a dream impossible of fulfilment that some similar method of self-organisation should draw other classes into the work.

Coming now to the measures designed to relieve the hospital of some part of its work or financial burden, or both, we have the establishment of pay pavilions. This has been advocated by Sir Henry Burdett in a scheme which he put forward in his speech at Leicester. His proposal is that such pay pavilions should be built by the hospital and in direct connection with it, the capital being borrowed from the State at 3 per cent., and 1 per cent. added for a sinking fund. Any proposal coming with the authority of Sir Henry Burdett commands the greatest consideration. But would the hospital entering on this enterprise not run the risk of adding to its own burdens? If the pay pavilion proved a business success, good and well. But if its success went no further than making ends meet, there would remain the burden of the annual interest, and there might even be in addition to this a loss on the year's working. It would be better, surely, that such homes should be established apart from the voluntary hospital. There is great need of them in all centres of population. A centre like Newcastle could at a reasonable estimate employ a home with fifty to a hundred beds, and it would to some extent relieve the general hospital in the pressure of its work.

A still more difficult problem is the co-ordination of the hospitals in a geographical area. It is difficult to define the precise manner in which hospitals which experience little pressure could be utilised to relieve those in which the pressure is great. Apart from the practical hindrances there is the sentimental one. So long as hospitals enjoy their present independence they are in some sense rivals, and it would need the pressure of an outside authority to make such co-operation possible.

The utilisation of the Poor-law hospitals presents still greater obstacles. It is only a very few such hospitals, and these in the great centres, which are so constructed and equipped as to be capable of serving as up-to-date general hospitals. To be available two changes are necessary—the provision of a medical and surgical staff and their still further disassociation from the stigma and disability of pauperism. Of these changes the latter would prove the more difficult of accomplishment.

It must be confessed that none of these proposals for increasing the resources or relieving the hospitals of some part of their work have either the attraction of novelty or the assurance of success. In their struggle to make both ends meet hospital managers have turned to every quarter, and all possible expedients have been considered and exploited. It is not unlikely that in the end the position will be forced by the necessity not so much of obtaining income as of finding funds for capital outlay. In no long time the deficiency of hospital accommodation throughout the country will make itself so apparent as to render the finding of some solution of the difficulty imperative. The data do not exist for an exact calculation of the number of beds available in relation to population. I have endeavoured to arrive at some determination in the case of the combined counties of Northumberland and Durham. Counting, so far as I have been able to ascertain, all available beds, the proportion cannot amount to 1 in 1,000 of the population. This is less than half of what in Germany and France is accepted as a minimum. Especially in the present state of public feeling I think it would be vain to look to voluntary effort to supply the deficiency. The ability of the philanthropic public as well as their patience, and the endurance of those who made themselves responsible for the effort, would be soon exhausted. The only possible alternative would seem to be a subvention from public funds, which would require an Act of Parliament, and would necessarily entail a radical change in the position of the hospitals. But as in the case of the higher education, a subvention from the State, the Municipality, or the County Council would not necessarily destroy their voluntary character. It would greatly limit it, undoubtedly. Power would have to be conferred, say, on the County Borough and County Councils to aid the hospitals, especially in the matter of capital outlay, by the application of a fund possibly ear-marked for the purpose. It could be either a fund locally raised by the County Borough or County Council, or provided by the Exchequer after the manner of the so-called residue under Section 1 of the Local Taxation (Customs and Excise) Act, 1890. To obtain such a grant or subvention—and its use need not be confined to capital outlay—the application on the part of the hospital would be followed by inquiry through some constituted authority into the necessities of the case. It is not impossible that the existence of such a resource might encourage rather than deter from voluntary effort.

If, on the other hand, the carrying-out of this or any similar proposal should hasten the handing over of the hospitals to State or municipal support and control, it will only be because the current has set inevitably in that direction. It may be that all the makeshifts we can adopt—and confessedly they are makeshifts—will succeed only in building some half-way house in which nobody will long wish to abide. If that be so, I sincerely trust that the fate of the hospitals will be to be handed over to the care of the local authorities. The more completely the principles of our local self-government are carried out in such a transference the better; and taking my courage in both my hands I would venture to put forward the following as a rough sketch of a possible ultimate solution. I would begin by dividing the country into hospital areas. Each area would have at least one hospital of the first rank, which would be the centre of the system, though not necessarily the geographical centre. *A hospital authority* would be constituted, which would have the control of those hospitals within the area which were maintained by public money. This authority would be appointed by the County Borough Councils and County Councils of the area. Each hospital would have its own managing committee, some of whose members would be *ex officio*, the remainder nominated by the hospital authority. This managing committee would be of moderate size; an ideal committee would not exceed nine members. Upon it I should have the Mayor of the city or borough, or the Chairman of the Urban or District Council; and the leading clergy, or ministers of the Free Churches, and the heads of the chief educational institutions and colleges, and the working classes would be represented. The spheres of these managing committees and of the hospital authorities would require precise definition. The funds required should be provided partly by a borough and county rate, partly by a subvention from the State.

It is often a matter of speculation whether under such a *régime* the stream of philanthropic donations and bequests would entirely cease. I think such an issue highly improbable. Hospitals would always remain centres of popular interest, and their needs would include many which could hardly be reached by public funds. It is not the experience of institutions devoted to the higher education that because they are largely self-supporting and enjoy grants in aid they are therefore forgotten by the public-spirited donor or testator. In America, where hospital administration and support are found in every possible variety, we know how frequently the hospital is founded or endowed by the gifts of the wealthy.

I should be very specially solicitous that in any future settlement of the hospitals a way of escape should be found from two possible calamities. I should consider it no less than a calamity that they should fall unreservedly into the hands of the State and should risk being strangled by the bureaucratic government and red tape which such a fate would threaten. It seems to me that this could only be escaped by the faithful carrying-out of the principles of local self-government. The other evil that I should fear which hospitals such as I am imagining might foster if they were made entirely free would be the tendency to pauperisation of those who received their benefits. The classes from which patients would be drawn would probably escape payment of any part of the rate levied for the maintenance of the hospitals. Many of them would nevertheless be able to pay in part or even in whole for their treatment—or, if not they themselves, their Friendly Societies or the Insurance Commission. The hospital should, in fact, be conducted on business lines, and should expect payment from all who, themselves or their representatives, can pay. The fit objects for charity would be charitably maintained and treated as heretofore. Co-ordination among the hospitals of the area would be possible, being under the direction of one authority, who would have first-hand knowledge of the special wants of the district.

